# Mental Health of COVID-19 Survivors at 6 and 12 Months Postdiagnosis: A Cohort Study

**DOI:** 10.3389/fpsyt.2022.863698

**Published:** 2022-04-08

**Authors:** Xin Huang, Lin Liu, Buzohre Eli, Jingyi Wang, Yaru Chen, Zhengkui Liu

**Affiliations:** ^1^Chinese Academy of Science (CAS) Key Laboratory of Mental Health, Institute of Psychology, Beijing, China; ^2^Department of Psychology, University of Chinese Academy of Sciences, Beijing, China; ^3^Jinyintan Hospital, Wuhan, China

**Keywords:** COVID-19, hospitalized survivors, PTSD, depression, anxiety

## Abstract

**Objective:**

As COVID-19 persists around the world, it is necessary to explore the long-term mental health effects in COVID-19 survivors. In this study, we investigated the mental health outcomes of survivors of COVID-19 at 6 and 12 months postdiagnosis.

**Methods:**

Posttraumatic stress disorder (PTSD checklist for the DSM-5, PCL-5), depression (PHQ-9), anxiety (Generalized Anxiety Disorder Scale, GAD-7), resilience (Connor-Davidson Resilience Scale, CD-RISC-10), perceived social support (PSSS), personality traits (Chinese Big Five Personality Inventory-15, CBF-PI-15), and sociodemographic information were examined among 511 survivors of COVID-19 (48.1%, females; *M*_age_ = 56.23 years at first assessment) at 6 and 12 months postdiagnosis. The data were analyzed with Wilcoxon signed rank tests and multivariable logistic regression models.

**Results:**

The prevalence of anxiety, depression, and posttraumatic stress disorder (PTSD) at 6 and 12 months after diagnosis was 13.31% and 6.26%; 20.35% and 11.94%; and 13.11% and 6.07%, respectively. The risk factors for all symptoms were as follows: higher neuroticism; lower openness, extraversion, agreeableness, and resilience; greater life disruptions due to COVID-19; poorer living standards; and increased symptoms of PTSD or depression at 6 months postdiagnosis.

**Conclusion:**

The mental health of COVID-19 survivors improved between 6 and 12 months postdiagnosis. Mental health workers should pay long-term attention to this group, especially to survivors with risk factors.

## Introduction

As of February 15, 2022, the global pandemic of COVID-19—an infectious disease caused by severe acute respiratory syndrome coronavirus 2 (SARS-CoV-2)—has resulted in more than 410.6 million confirmed cases with more than 5.8 million deaths (1). Since December 2019, people around the world have continued to fight against this new disease for more than 2 years; however, we might have to live with COVID-19 for a long period of time.

For patients surviving COVID-19 infection, experiencing COVID-19 might cause substantial trauma (2). After being diagnosed with COVID-19, there was much mental suffering for patients (3). Previous studies reported that 13.2–96.2%, 21.0–33.7%, and 16.4–34.72% of patients with COVID-19 exhibited posttraumatic stress disorder (PTSD), depression, and anxiety symptoms, respectively (2, 4–6). These studies were conducted within 3 months after the participants received confirmation of their COVID-19 infection, which revealed the immediate mental impact of COVID-19. Regarding the long-term effects of COVID-19 on survivors, these individuals continued a physical recovery phase after leaving the hospital (7, 8) and reported physical and psychological sequelae (9, 10). A study in Norway found that 9.5% of hospitalized patients reported PTSD symptoms at a median of 116 days after COVID-19 onset (11). A study including 251 participants in Brazil found that 81 (32.27%) had a positive screening for anxiety/depression 3 months following hospital discharge (12). A study in China found that 6 months after discharge from the hospital, 23% (367 of 1,617) of patients reported depression or anxiety (2). Another study investigating 152 patients 6 months after discharge also reported poor mental health compared to baseline conditions (13). In summary, survivors of COVID-19 continue to exhibit mental health problems after discharge. Moreover, the duration of the pandemic might have chronic effects on mental health. Thus, longitudinal assessments are essential for evaluating the long-term effects on the mental health of COVID-19 survivors, especially at periods of time longer than 6 months after discharge. We intended to explore the long-term mental health of COVID-19 survivors and compare the differences in their mental health over time.

Studies about previous pandemics, such as severe acute respiratory syndrome (SARS) and Middle East respiratory syndrome (MERS), might remind us about how the mental status of survivors of a pandemic changes over time (14–16). Survivors might show relatively high psychological distress during the acute phase (17). Cheng et al. reported that 35% of 425 survivors expressed having anxiety or depressive symptoms at 1 month post-SARS infection (17), and although the severity of mental distress might decrease, it remains higher than that of the general public over the long term (18, 19). Chau et al. performed a systematic review and found that the prevalence of anxiety, depression, and PTSD among SARS survivors was 19, 20, and 28%, respectively, and the outcomes within the first 6 months and beyond 6 months postdischarge were not significantly different (14). However, considering that the duration of COVID-19 is much longer than that of SARS and MERS, we suspected that the mental health recovery process for survivors of COVID-19 would be much longer than that observed for the previous two pandemics.

Moreover, previous mental health status might affect subsequent mental health outcomes (20–22). Nikèeviæ et al. reported that health anxiety might predict subsequent generalized anxiety and depressive symptoms in United States residents (20). Wu et al. found that during the COVID-19 pandemic, depression symptoms could predict subsequent anxiety in university students (22). Previous PTSD severity was shown to predict follow-up PTSD severity (23). However, no study has examined the temporal associations of mental health outcomes in survivors of COVID-19. Therefore, we explored whether previous mental health symptoms could predict subsequent symptoms.

Some risk factors for mental health outcomes after disasters have been reported in literature, such as female sex, young age, lower socioeconomic status, higher education level, marital status (married for women and unmarried for men), greater exposure to the disaster, and lack of perceived social support (2, 24). We included the above factors in our investigation and examined whether the effects of these factors impacted COVID-19 survivors in the long term. Regarding personality factors, higher resilience might be conducive to maintaining mental health (25). Liu et al. examined the associations between the Big Five personality traits and stress and reported that higher neuroticism and extraversion were associated with higher levels of stress during the pandemic (26). Nikčević et al. found that neuroticism was positively correlated with generalized anxiety and depressive symptoms in the general public during the COVID-19 pandemic (20). The Big Five personality traits might have effects on the mental health of patients. Furthermore, a previous study found that body mass index (BMI) significantly changed in hospital staff during the COVID-19 pandemic (27), and there could be similar changes in patients. Therefore, we also intended to explore the effects of the Big Five personality traits and BMI on mental health in patients in this study. Moreover, at different phases of the pandemic, the impacts of the above factors might not be the same (18), so it was necessary to examine the effects of these factors at different time points.

In the current study, we aimed to examine the long-term mental health effects of COVID-19 infection and to identify predictors, as we investigated the mental health of COVID-19 survivors at 6 and 12 months after diagnosis. These patients were treated at Wuhan Jin-Yintan Hospital, which was the earliest medical center designated for fighting COVID-19 in China (28). We adopted depression, anxiety, and PTSD symptoms as indices for mental health. We included the socioeconomic and individual factors described above; specifically, severity the level of symptoms, ICU admission, relatives with COVID-19, and the level of life disruption by COVID-19 were included in the assessment of the effects of COVID-19 infection.

## Materials and Methods

### Procedures and Participants

After the COVID-19 outbreak, many patients were confirmed to be infected and accepted as inpatients in Wuhan Jin-Yintan Hospital. All 2,469 patients discharged between January 7 and May 29, 2020 were referred to Jin-Yintan Hospital for psychological and physical examinations. Our study was conducted through psychological examinations performed independently by a psychology graduate student and a nurse, who was trained by the graduate student to read the questions for participants who could not read. Additional details about the physical examination procedure are described elsewhere (7). The psychological investigations were conducted at two timepoints: T1, from July 24 to September 4, 2020, and T2, from December 16, 2020 to February 7, 2021. Informed consent was obtained from all participants at each timepoint. An online questionnaire was administered. The participants completed the questionnaire by themselves with the guidance of two nurses. For those without a smartphone, a paper questionnaire was provided; for illiterate individuals, a nurse read the questions and choices for them and completed the questionnaire based on their answers. Finally, 945 and 1,131 participants were included at T1 and T2, respectively, and 537 participated in both investigations. Then, 26 participants were excluded, which included 25 participants who answered the questionnaires in less than 200 s and one participant whose birthdate was inaccurate. Thus, 511 participants (at T1: sex: 246 females/265 males; age: *M* = 56.23, *SD* = 12.18) were included in the analysis. The research proposal was approved by the ethics review committee of the Institute of Psychology, Chinese Academy of Sciences.

### Measures

#### Demographic Variables

The basic demographic information included age, sex, BMI, educational level (primary school, junior high school, high school/technical secondary school, junior college/university, postgraduate, or above), living standard (low, below average, medium, or above average), and marital status (unmarried, married, divorced, remarried, or widowed). The variables related to how the participants were affected by COVID-19 were as follows: date of diagnosis, length of stay, severity level of symptoms associated with COVID-19 (none, mild, medium, or serious), ICU admission (no or yes), whether they had relatives confirmed to have COVID-19 (no or yes), and to what level their life had been disrupted by COVID-19 (none, mild, medium, and serious). The demographic variables were measured at T1.

#### Posttraumatic Stress Disorder

The PTSD checklist for the DSM-5 (PCL-5) was used at T1 and T2. The PCL-5 comprises 20 items that assess all DSM-5 PTSD symptoms. Each item was rated on a scale of 0–4 (0 = “none” to 4 = “severe”), and the total score could range from 0 to 80 (29). Higher scores indicated a more severe degree of PTSD, and scores above 33 indicated probable PTSD (29). The PCL-5 was shown to exhibit a strong reliability and validity (2). In the current study, Cronbach’s alpha values for the scale at T1 and T2 were 0.935 and 0.961, respectively.

#### Depression

The Chinese version of the 9-item Patient Health Questionnaire (PHQ-9) was used to assess depression symptoms at T1 and T2. Each item describes a specific depressive symptom rated on a scale of 0–4 (0 = “not at all” to 3 = “every day”), and total scores can range from 0 to 27 (30). The PHQ-9 has been shown to be valid and reliable and to have good diagnostic utility. Scores above 9 indicate a probable depression disorder (30). In the present study, Cronbach’s alpha values at T1 and T2 were 0.898 and 0.930, respectively.

#### Anxiety

The Generalized Anxiety Disorder Scale (GAD-7) was used to examine the severity of anxiety at T1 and T2. The items on the GAD-7 are rated on a four-point scale (0 = “not at all” to 3 = “every day”), and total scores can range from 0 to 21 (31). The GAD-7 was shown to have good reliability, validity and diagnostic utility. Scores above 9 indicate a probable anxiety disorder (31). In the present study, Cronbach’s alpha values at T1 and T2 were 0.932 and 0.959, respectively.

#### Resilience

The abbreviated version of the Connor-Davidson Resilience Scale (CD-RISC-10) was used to assess resilience at T1 and T2. The CD-RISC-10 is a 10-item self-report scale with good psychometric properties. The items are scored on a five-point scale from 0 to 4 (0 = “none” to 4 = “always”) (32). The total score can range from 12 to 84, and higher total scores indicate a higher level of resilience. In this study, Cronbach’s alpha values at T1 and T2 were 0.967 and 0.982, respectively.

#### Perceived Social Support

The Chinese version of the perceived social support scale (PSSS) was administered at T1 and T2 to measure perceived social support from significant others, family members, and friends. The PSSS comprises 12 items rated from 1 to 7 (1 = “very strongly disagree” to 7 = “very strongly agree”) (33). The total score can range from 12 to 84, and higher scores indicate higher perceived social support. The PSSS has been shown to have strong psychometric properties (34). In this investigation, Cronbach’s alpha values at T1 and T2 were 0.955 and 0.967, respectively.

#### Big Five Personality

The Chinese Big Five Personality Inventory-15 (CBF-PI-15) was used to assess the Big Five personality traits at T2. The CBF-PI-15 is a very short version of the Chinese Big Five Personality Inventory (CBF-PI) and comprises 15 items answered on a six-point scale ranging from 1 (“disagree strongly”) to 6 (“agree strongly”) (35). The CBF-PI-15 has been shown to have good reliability and validity. In this investigation, Cronbach’s alpha values ranged from 0.836 (agreeableness) to 0.895 (conscientiousness), with the exception of 0.304 for openness. We included the openness dimension in the analysis to maintain experimental integrity.

### Data Analysis

Demographic characteristics are presented as the mean (*M*) and standard deviation (SD) for continuous variables (see Table 1) and as numbers with percentages for categorical variables (see Table 2). Mental health characteristics are expressed as the mean and SD. Wilcoxon signed rank tests were adopted to examine the changes in the variables between T1 and T2. Then, all the predictors were included in multivariable logistic regression models of the mental health outcomes. In particular, aspects of mental health at T1 were examined as predictors for outcomes at T2. Analyses were performed with SPSS 26.

**TABLE 1:** Pearson correlation of the variables of the participants.

		Mean (*SD*)	1	2	3	4	5	6	7	8	9	10	11	12	13	14	15	16	17	18	19	20	21
1	Age	56.23 (12.18)	1																				
2	Days between discharge and T1	151.79 (24.25)	–0.07	1																			
3	Days between discharge and T2	307.35 (22.06)	–0.04	0.88**	1																		
4	Length of stay (days)	36.01 (23.08)	0.08	–0.67**	–0.71**	1																	
5	BMI at T1	24.64 (3.40)	–0.04	0.03	0.03	–0.01	1																
6	BMI at T2	25.07 (3.41)	0.01	0.03	0.03	–0.02	0.92**	1															
7	Resilience at T1	26.06 (12.43)	0.03	0.10*	0.06	–0.05	0.01	0.02	1														
8	Resilience at T2	24.50 (12.09)	–0.11*	0.12**	0.11*	–0.07	–0.08	–0.08	0.19**	1													
9	Social support at T1	58.85 (20.30)	0	0.18**	0.18**	–.010*	–0.01	–0.02	0.54**	0.19**	1												
10	Social support at T2	60.21 (16.51)	–0.11*	0.12**	0.10*	–0.02	–0.01	–0.01	0.15**	0.51**	0.33**	1											
11	Neuroticism	9.09 (4.04)	0	–0.08	–0.08	0.04	–0.03	–0.03	–0.03	0.11*	–0.03	0.12**	1										
12	Openness	9.06 (4.60)	–0.21**	0.07	0.07	–0.04	–0.06	–0.09*	0.05	0.35**	0.07	0.48**	0.12**	1									
13	Conscientiousness	12.52 (4.1)	–0.20**	0.04	0.02	0.04	–0.07	–0.09	0.12**	0.56**	0.21**	0.69**	0.23**	0.58**	1								
14	Extraversion	10.05 (3.13)	–0.04	0.08	0.14**	–0.08	0.08	0.07	0.02	–0.17**	0.06	–0.05	–0.30**	–0.06	–0.22**	1							
15	Agreeableness	13.67 (3.96)	–0.07	0.03	–0.02	0.02	–0.02	–0.01	0.21**	0.60**	0.24**	0.68**	0.26**	0.35**	0.69**	–0.23**	1						
16	PTSD at T1	16.33 (15.24)	0.09*	–0.13**	–0.10*	0.07	0	0.01	–0.18**	–0.15**	–0.16**	–0.18**	0.27**	–0.14**	–0.08	–0.16**	–0.08	1					
17	PTSD at T2	10.99 (12.08)	0.01	–0.07	–0.07	0.07	0.04	0.03	–0.17**	–0.12**	–0.12**	–0.17**	0.30**	–0.08	–0.06	–0.16**	–0.12**	0.54**	1				
18	Depression at T1	5.73 (5.78)	0.05	–0.09*	–0.07	0.06	0.04	0.05	–0.20**	–0.12**	–0.18**	–0.16**	0.21**	–0.12**	–0.10*	–0.12**	–0.06	0.81**	0.47**	1			
19	Depression at T2	3.70 (4.78)	–0.01	–0.08	–0.07	0.06	0.03	0.02	–0.18**	–0.14**	–0.11*	–0.17**	0.27**	–0.10*	–0.09*	–0.11*	–0.13**	0.48**	0.86**	0.51**	1		
20	Anxiety at T1	3.96 (5.05)	–0.01	–0.10*	–0.07	0.03	0.03	0.04	–0.18**	–0.06	–0.18**	–0.13**	0.27**	–0.09*	–0.03	–0.16**	–0.04	0.77**	0.48**	0.77**	0.43**	1	
21	Anxiety at T2	2.60 (4.10)	–0.03	–0.08	–0.06	0.03	0.02	0.01	–0.16**	–0.13**	–0.11*	–0.19**	0.32**	–0.11**	–0.05	–0.16**	–0.13**	0.45**	0.81**	0.43**	0.87**	0.44**	1

*Correlation was significant at the 0.05 level (two-tailed).

**Correlation was significant at the 0.01 level (two-tailed).

*T1, the first survey time point; T2, the second survey time point; BMI, body mass index. Variables measured twice are marked with T1 and T2 to distinguish the time, e.g., BMI at T2 was the BMI measured at T2. Big Five traits (11–15) were measured at T2. Other variables without marks were measured at T1.*

**TABLE 2:** Sociodemographic characteristics and mental health of the participants.

Characteristics		Number	PTSD at T1	PTSD at T2	Depression at T1	Depression at T2	Anxiety at T1	Anxiety at T2
**Sex**								
	Female	246 (48.14%)	18.87 (16.55)	11.82 (0.83)	6.40 (6.07)	3.98 (5.14)	4.93 (5.56)	2.95 (4.53)
	Male	265 (51.86%)	13.97 (13.51)	10.23 (0.68)	5.11 (5.44)	3.45 (4.41)	3.05 (4.35)	2.28 (3.63)
**Education**								
	Primary school	43 (8.41%)	16.47 (13.31)	12.95 (17.52)	4.95 (4.87)	3.37 (5.57)	4.19 (4.40)	3.02 (5.93)
	Junior high school	145 (28.38%)	16.68 (15.66)	10.59 (12.66)	5.47 (5.72)	3.44 (4.89)	4.08 (5.24)	2.32 (4.03)
	High school/technical secondary school	170 (33.27%)	17.21 (16.28)	10.51 (10.62)	6.59 (6.28)	3.71 (4.53)	4.45 (5.37)	2.55 (3.65)
	Junior college/university	142 (27.79%)	15.23 (14.14)	11.49 (11.34)	5.37 (5.39)	4.18 (4.80)	3.26 (4.52)	2.89 (4.11)
	Postgraduate or above	11 (2.15%)	11.82 (14.67)	9.82 (9.47)	3.73 (5.98)	2.09 (3.21)	3.00 (6.02)	1.55 (2.88)
**Living standard**								
	Low	63 (12.33%)	26.19 (18.76)	16.33 (13.82)	8.70 (6.59)	5.17 (5.40)	6.78 (6.13)	3.65 (3.96)
	Below average	170 (33.27%)	16.31 (15.34)	11.52 (13.64)	5.78 (6.03)	3.88 (5.15)	4.35 (5.35)	2.92 (4.67)
	Medium	250 (48.92%)	14.43 (13.50)	9.68 (10.12)	5.07 (5.19)	3.33 (4.34)	3.12 (4.33)	2.22 (3.74)
	Above average	28 (5.48%)	11.25 (11.67)	7.50 (10.51)	4.68 (5.47)	2.61 (4.09)	2.68 (3.92)	1.64 (3.35)
**Marital status**								
	Unmarried	21 (4.11%)	21.48 (19.94)	12.57 (10.27)	8.29 (7.96)	5.19 (4.49)	5.29 (6.56)	3.1 (3.82)
	Married	397 (77.69%)	15.71 (14.76)	10.62 (11.98)	5.48 (5.57)	3.54 (4.77)	3.80 (5.04)	2.58 (4.2)
	Divorced	33 (6.46%)	17.91 (17.50)	11.64 (14.55)	6.85 (6.63)	4.00 (4.86)	4.55 (5.43)	3.03 (4.53)
	Remarried	8 (1.57%)	13.50 (19.08)	6.38 (12.14)	5.50 (6.14)	2.38 (5.21)	3.00 (4.75)	1.63 (4.21)
	Widowed	52 (10.18%)	18.38 (14.48)	13.50 (11.72)	5.98 (5.62)	4.33 (4.81)	4.44 (4.24)	2.42 (3.16)
**Order of severity**								
	None	16 (3.13%)	18.69 (19.64)	13.06 (11.80)	6.13 (6.54)	3.38 (3.54)	7.06 (8.30)	2.63 (3.03)
	Mild	228 (44.62%)	14.45 (13.53)	9.36 (10.98)	4.90 (5.10)	3.03 (4.13)	3.63 (4.69)	2.17 (3.69)
	Medium	154 (30.14%)	15.68 (15.49)	10.62 (11.04)	5.82 (6.21)	3.81 (4.55)	3.86 (5.03)	2.76 (4.14)
	Serious	113 (22.11%)	20.67 (16.71)	14.50 (14.69)	7.23 (6.11)	4.97 (6.06)	4.33 (5.09)	3.25 (4.85)
**ICU admission**								
	No	283 (55.38%)	17.84 (16.35)	12.31 (0.76)	6.40 (6.21)	4.25 (5.18)	4.23 (5.31)	2.95 (4.38)
	Yes	228 (44.62%)	14.45 (13.53)	9.36 (0.73)	4.90 (5.10)	3.03 (4.13)	3.63 (4.69)	2.17 (3.69)
**Relatives with COVID-19**								
	No	268 (52.45%)	13.94 (13.36)	9.93 (12.25)	4.74 (5.07)	3.26 (4.58)	3.40 (4.62)	2.43 (4.22)
	Yes	243 (47.55%)	18.96 (16.70)	12.17 (11.79)	6.83 (6.31)	4.19 (4.95)	4.58 (5.42)	2.79 (3.97)
**Life disturbed by COVID-19**								
	None	72 (14.09%)	8.36 (10.36)	6.94 (12.05)	3.11 (4.46)	2.17 (4.32)	2.54 (5.03)	1.88 (3.91)
	Mild	183 (35.81%)	12.46 (11.16)	8.43 (8.61)	4.21 (4.25)	2.75 (3.47)	2.78 (3.60)	1.96 (3.42)
	Medium	137 (26.81%)	18.71 (15.47)	10.85 (10.14)	6.58 (6.12)	3.71 (4.43)	4.53 (5.28)	2.41 (3.72)
	Serious	119 (23.29%)	24.36 (18.36)	17.55 (15.71)	8.68 (6.64)	6.09 (6.13)	5.97 (5.92)	4.24 (5.08)

*Data were n (%) or the mean (SD); bold indicates p < 0.05. ICU, intensive care unit; T1, the first survey time point; T2, the second survey time point; PTSD, posttraumatic stress disorder. Order of severity—severity level of the symptoms associated with COVID-19; relatives with COVID-19—whether the participant had relatives confirmed to have COVID-19; life disturbed by COVID-19—to what level the participant’s life was disrupted by COVID-19. Variables measured twice are marked with T1 and T2 to distinguish the time, e.g., PTSD at T2 was PTSD measured at T2.*

## Results

### Participants

On average, the patients participated in the investigation for 6 months (T1: *M* = 187.62 days, *SD* = 18.75) or 12 months (T2: *M* = 343.19 days, *SD* = 16.75) after the date of diagnosis. The sociodemographic and personality characteristics of the sample are displayed in Tables 1, 2. The education levels of most of the participants were high school/technical secondary school (33.27%), followed by junior high school (28.38%) and junior college/university (27.79%); only a few participants had a primary school education (8.41%) or postgraduate education or above (2.15%). Most participants perceived that they lived at medium (48.92%) or below average (33.27%) living standards. The majority were married (77.69%) or widowed (10.18%).

### COVID-19 Experience

The mean length of stay in the hospital was 36.01 days (*SD* = 23.08). The patients reported various levels of severity: 22.11% reported serious symptoms; 30.14% reported medium symptoms; 44.62% reported mild symptoms; and only 3.13% reported no symptoms. Approximately half (44.62%) of the patients were admitted to the ICU, and 47.55% had relatives with COVID-19. The lives of most patients were disrupted by COVID-19 (85.91%); more precisely, 35.81% had mild, 26.81% had medium, and 23.29% had serious disruptions. These discharged patients were severely affected by COVID-19.

### The Effects of Time on Mental Health and Resilience, Social Support, and Body Mass Index

**FIGURE 1:**
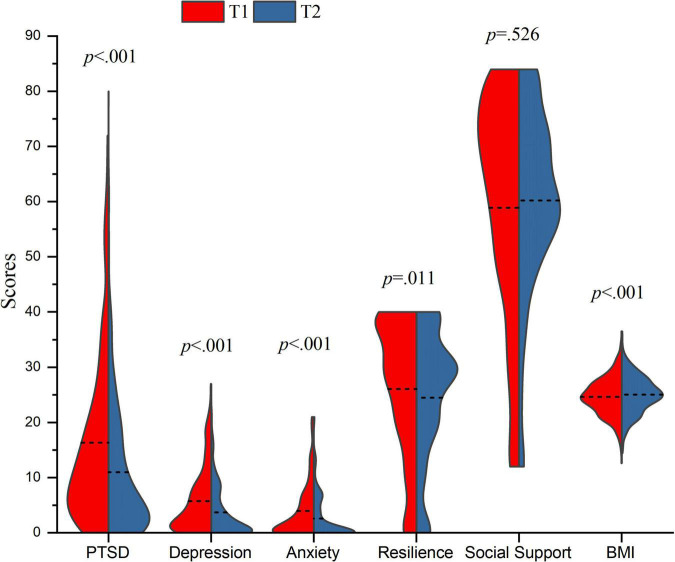
Temporal changes of PTSD, depression, anxiety, resilience, social support, and BMI. related-samples Wilcoxon signed rank test were conducted on PTSD, depression, anxiety, resilience and social support due to the non-normality of data. The paired samples *t*-test was conducted on BMI based on the normality of data. The horizontal dash lines were used to indicate the mean value.

As shown in Tables 1, 2 and Figure 1, at T1 and T2, 67 (13.11%) and 31 (6.07%) patients, respectively, had PTSD scores above 33; 104 (20.35%) and 61 (11.94%) had depression scores above 9; and 68 (13.31%) and 32 (6.26%) had anxiety scores above 9. The mean PTSD, depression, and anxiety scores at T1 and T2 were 16.33 (*SD* = 15.24) and 10.99 (*SD* = 12.08), 5.73 (*SD* = 5.78) and 3.70 (*SD* = 4.78), and 3.96 (*SD* = 5.05) and 2.60 (*SD* = 4.10), respectively, all of which showed significant decreases (*p* < 0.001). Interestingly, the mean BMI at T1 and T2 was 24.64 (*SD* = 3.40) and 25.07 (*SD* = 3.14), respectively, which showed a significant increase (*p* < 0.001). Moreover, the mean resilience scores (T1: *M* = 26.06, *SD* = 12.43; T2: *M* = 24.50, *SD* = 12.09) significantly changed (*p* = 0.011), while social support (T1: *M* = 58.85, *SD* = 20.30; T2: *M* = 60.21, *SD* = 16.51) did not significantly change (*p* = 0.526).

### Predictors of Posttraumatic Stress Disorder, Depression, and Anxiety Symptoms at T1 and T2

The results of multivariate logistic regression for PTSD, depression, and anxiety are shown in Table 3. At T1, higher neuroticism and serious [odds ratio (OR) = 4.18, 95% confidence interval (CI) = 1.14–15.41, *p* = 0.032] life disruptions from COVID-19, compared to no influence, added to the probability of having PTSD symptoms. Higher openness (OR = 0.90, 95% CI = 0.83–0.97, *p* = 0.005) and a medium living standard compared to a low living standard (OR = 0.36, 95% CI = 0.15–0.85, *p* = 0.020) reduced the probability of having PTSD symptoms. Regarding depression, at T1, higher neuroticism (OR = 1.07, 95% CI = 1.00–1.15, *p* = 0.048), a high school/technical secondary school education compared to a primary school education (OR = 3.46, 95% CI = 1.24–9.66, *p* = 0.018), and medium (OR = 3.95, 95% CI = 1.40–11.13, *p* = 0.009) and serious (OR = 4.51, 95% CI = 1.56–13.05, *p* = 0.005) levels of life disruption from COVID-19, compared to no disruptions, increased the probability of depression. Higher resilience (OR = 0.96, 95% CI = 0.94–0.99, *p* = 0.003) and openness (OR = 0.92, 95% CI = 0.86–0.98, *p* = 0.013) reduced the likelihood of depression. Regarding anxiety, higher neuroticism (OR = 1.12, 95% CI = 1.03–1.22, *p* = 0.008) increased the probability of anxiety. Higher extraversion (OR = 0.89, 95% CI = 0.81–0.98, *p* = 0.019), higher openness (OR = 0.97, 95% CI = 0.95–0.99, *p* = 0.002), and medium living standards, compared to low living standards (OR = 0.25, 95% CI = 0.11–0.59, *p* = 0.002), reduced the likelihood of anxiety.

**TABLE 3:** Multivariate logistic regressions of PTSD, depression, and anxiety at T1 and T2.

Characteristics	PTSD at T1		Depression at T1		Anxiety at T1		PTSD at T2		Depression at T2		Anxiety at T2	
	*p-*value	OR (95% CI)	*p-*value	OR (95% CI)	*p-*value	OR (95% CI)	*p-*value	OR (95% CI)	*p-*value	OR (95% CI)	*p-*value	OR (95% CI)
Neuroticism	**<0.001**	**1.20 (1.09, 1.33)**	**0.048**	**1.07 (1.00, 1.15)**	**0.008**	**1.12 (1.03, 1.22)**	**<0.001**	**1.49 (1.18, 1.88)**	**<0.001**	**1.30 (1.15, 1.46)**	**<0.001**	**1.49 (1.21, 1.84)**
Openness	**0.032**	**0.91 (0.83, 0.99)**	**0.013**	**0.92 (0.86, 0.98)**	**0.011**	**0.90 (0.83, 0.98)**	0.451	0.94 (0.80, 1.10)	**0.048**	**0.91 (0.82, 1.00)**	0.104	0.89 (0.78, 1.02)
Extraversion	0.074	0.89 (0.78, 1.01)	0.457	0.97 (0.88, 1.06)	**0.034**	**0.88 (0.79, 0.99)**	0.275	0.88 (0.70, 1.11)	0.154	0.90 (0.78, 1.04)	**0.023**	**0.78 (0.63, 0.97)**
Agreeableness	0.085	0.91 (0.81, 1.01)	0.979	1.00 (0.91, 1.09)	0.389	0.95 (0.86, 1.06)	**<0.001**	**0.59 (0.45, 0.77)**	**0.002**	**0.80 (0.70, 0.92)**	**0.001**	**0.69 (0.55, 0.85)**
Resilience	0.129	0.98 (0.95, 1.01)	**0.003**	**0.96 (0.94, 0.99)**	0.180	0.98 (0.95, 1.01)	0.279	1.03 (0.98, 1.09)	0.278	0.98 (0.95, 1.02)	0.212	1.04 (0.98, 1.10)
PTSD at T1	Not applicable		Not applicable		Not applicable		**0.046**	**1.06 (1.00, 1.12)**	0.696	1.01 (0.97, 1.04)	0.853	1.00 (0.96, 1.05)
Depression at T1	Not applicable		Not applicable		Not applicable		0.341	1.07 (0.93, 1.24)	**0.007**	**1.15 (1.04, 1.26)**	0.117	1.12 (0.97, 1.30)
Education												
Postgraduate or above	0.760	0.59 (0.02, 17.61)	0.532	0.39 (0.02, 7.33)	0.746	1.53 (0.12, 20.37)	–	–	–	–	–	–
Junior college/ University	0.225	2.20 (0.62, 7.84)	0.303	1.81 (0.59, 5.56)	0.769	1.21 (0.34, 4.28)	0.824	0.80 (0.11, 5.89)	0.061	4.25 (0.94, 19.23)	0.793	1.27 (0.21, 7.71)
High school/technical secondary school	0.254	1.94 (0.62, 6.02)	**0.018**	**3.46 (1.24, 9.66)**	0.303	1.79 (0.59, 5.43)	0.200	0.34 (0.07, 1.77)	0.415	1.78 (0.45, 7.14)	0.082	0.24 (0.05, 1.20)
Junior high school	0.568	1.40 (0.44, 4.44)	0.200	1.96 (0.70, 5.52)	0.316	1.75 (0.59, 5.21)	0.807	0.81 (0.15, 4.33)	0.315	2.05 (0.51, 8.27)	0.352	0.47 (0.10, 2.29)
Primary school	Ref	Ref	Ref	Ref	Ref	Ref	Ref	Ref	Ref	Ref	Ref	Ref
Living standard												
Above-average	0.262	0.35 (0.06, 2.18)	0.647	0.73 (0.19, 2.77)	0.150	0.20 (0.02, 1.78)	0.821	0.72 (0.04, 12.69)	0.137	0.14 (0.01, 1.86)	0.625	1.93 (0.14, 26.64)
Medium	**0.020**	**0.36 (0.15, 0.85)**	**0.042**	**0.46 (0.21, 0.97)**	**0.002**	**0.25 (0.11, 0.59)**	0.094	0.26 (0.05, 1.26)	0.314	0.60 (0.22, 1.63)	0.893	0.91 (0.22, 3.70)
Below average	0.117	0.51 (0.22, 1.19)	0.106	0.54 (0.25, 1.14)	0.078	0.50 (0.23, 1.08)	0.549	1.49 (0.40, 5.53)	0.993	1.01 (0.38, 2.66)	0.525	1.52 (0.42, 5.57)
Low	Ref	Ref	Ref	Ref	Ref	Ref	Ref	Ref	Ref	Ref	Ref	Ref
Life disturbed by COVID-19												
Serious	**0.032**	**4.18 (1.14, 15.41)**	**0.005**	**4.51 (1.56, 13.05)**	0.118	2.42 (0.80, 7.30)	0.612	1.68 (0.23, 12.45)	0.909	0.93 (0.27, 3.22)	0.398	2.37 (0.32, 17.50)
Medium	0.076	3.22 (0.89, 11.73)	**0.009**	**3.95 (1.40, 11.13)**	0.141	2.23 (0.77, 6.45)	0.463	0.44 (0.05, 4.00)	0.409	0.59 (0.16, 2.09)	0.616	0.58 (0.07, 4.83)
Mild	0.788	0.83 (0.22, 3.18)	0.778	0.86 (0.30, 2.49)	0.291	0.55 (0.18, 1.67)	0.463	0.46 (0.06, 3.70)	0.108	0.37 (0.11, 1.25)	0.900	0.89 (0.13, 5.97)
None	Ref	Ref	Ref	Ref	Ref	Ref	Ref	Ref	Ref	Ref	Ref	Ref

*The patients with anxiety scores > 9 were considered to have a mental disorder; the patients with depression scores > 9 were considered to have a mental disorder; and the patients with PTSD scores > 33 were considered to have a mental disorder. Bold indicates p < 0.05. All the predictors were included in the multivariate logistic regressions (these three predictors, PTSD at T1, depression at T1, and anxiety at T1, were only included in the models of PTSD at T2, depression at T2, and anxiety at T2); for brevity, only predictors with significant effects are presented. OR, odds ratio; Ref, reference; CI, confidence interval; PTSD, posttraumatic stress disorder; T1, the first survey time point; T2, the second survey time point. Variables measured twice are marked with T1 and T2 to distinguish the time, e.g., PTSD at T2 was PTSD measured at T2; reference category—no PTSD symptoms for the PTSD model, no depression symptoms for the depression model, and no anxiety symptoms for the anxiety model.*

At T2, higher neuroticism (OR = 1.49, 95% CI = 1.18–1.88, *p* < 0.001) and PTSD scores at T1 (OR = 1.06, 95% CI = 1.00–1.12, *p* = 0.046) increased the probability of having PTSD symptoms; higher agreeableness (OR = 0.59, 95% CI = 0.45–0.77, *p* < 0.001) reduced the probability of having PTSD symptoms. Regarding depression, higher neuroticism (OR = 1.30, 95% CI = 1.15–1.46, *p* < 0.001) and depression scores at T1 (OR = 1.15, 95% CI = 1.04–1.26, *p* = 0.007) increased the probability of depression; higher openness (OR = 0.91, 95% CI = 0.82–1.00, *p* = 0.048) and higher agreeableness (OR = 0.80, 95% CI = 0.70–0.92, *p* = 0.002) reduced the likelihood of depression. Regarding anxiety, higher neuroticism (OR = 1.35, 95% CI = 1.18–1.56, *p* < 0.001) increased the probability of anxiety, while higher extraversion (OR = 0.78, 95% CI = 0.63–0.97, *p* = 0.023) and higher agreeableness (OR = 0.69, 95% CI = 0.55–0.85, *p* = 0.001) reduced that probability.

## Discussion

This study aimed to investigate the mental health of survivors of COVID-19 in the long term as the pandemic continues to persist, examine whether the levels of depression, anxiety, and PTSD would decrease between 6 (T1) and 12 (T2) months after diagnosis and identify predictors of these mental health problems at T1 and T2. Our initial finding was that as COVID-19 continues to persist, the prevalence of anxiety, depression, and PTSD in COVID-19 survivors at 6 and 12 months after diagnosis were 13.31 and 6.26%, 20.35 and 11.94%, and 13.11 and 6.07%, respectively; all of these showed significant decreases and a trend of mental improvement. Moreover, higher neuroticism, lower openness, extraversion, agreeableness, and resilience, greater life disruptions due to COVID-19, poorer living standards and higher PTSD and depression scores at T1 were risk factors for mental health problems.

### The Effects of Time on Mental and Physical Health

We found that depressive, anxiety, and PTSD symptoms of COVID-19 survivors discharged from a representative hospital treating patients with COVID-19 (Wuhan Jin-Yintan Hospital) decreased significantly between T1 and T2. This result was not consistent with a previous study on SARS (14), which reported that the prevalence of these outcomes in the first 6 months postdischarge and beyond was not significantly different. This might be because SARS lasted for only a short time (from November 2002 to July 2003) (18), while COVID-19 has continued to persist for a long time (starting in December 2019 and not yet ending) (36). Survivors remain under the threat of COVID-19 after discharge from the hospital, and thus, the recovery of their mental health might be slower. This possibility can be supported by the following evidence. First, at 1 month post-SARS, 35% of the survivors expressed having anxiety or depressive symptoms (17), which was higher than the prevalence reported by Chau et al. showing mental health recovery between the acute and postacute phases in SARS survivors. However, the prevalence of anxiety, depression, and PTSD at 6 months postdiagnosis in the current study was similar to those in the acute phase, which was 16.4, 21.0, and 13.2%, respectively (2). The long duration of COVID-19 might have prolonged the acute phase. Second, although the prevalence of anxiety, depression, and PTSD at 6 months after diagnosis in the current study was seemingly less serious than the 19, 20, and 28% prevalence rates in SARS survivors reported by Chau et al. (14), there was still a significant decline in anxiety, depression, and PTSD symptoms in COVID-19 survivors between 6 and 12 months after diagnosis. Our findings revealed that there might be different features of the prevalence and recovery process of mental health problems between COVID-19 and SARS, which still needs further study. We recommend that more attention be paid to the mental health of COVID-19 survivors over the long term, although their physical health may be protected.

We also found that resilience significantly decreased, which might be the result of the pressures associated with the ongoing pandemic (37). This can encourage mental health workers to conduct effective interventions to enhance people’s resilience as the pandemic continues. Social support did not significantly vary between T1 and T2, showing the relative stability of social relationships, without much change due to COVID-19.

Interestingly, the BMI of survivors significantly increased. This might have been because the stress of COVID-19 increased the desire to eat (38). Considering that obesity is a risk factor for severe disease and mortality in people with COVID-19 infection (39), health workers should be careful not to let patients overeat.

### Sociodemographic Factors Related to Mental Health Outcomes

This study found that lower education, enduring greater life disruptions due to COVID-19, and having poorer living standards were associated with increases in poor mental health outcomes. Guo et al. also reported that a low educational level was a risk factor for anxiety (40); however, the effect of education in our study was weak, which might need further study, so we do not consider it as a major predictor. Those whose lives were more seriously disrupted by COVID-19 were more likely to report PTSD and depression symptoms at 6 months postdiagnosis, which was consistent with previous studies (2), showing a dose-dependent effect. This impact could be explained by the level of trauma caused by COVID-19 (2), as those having greater life disruptions and relatives with COVID-19 might experience more psychological trauma. However, as time passed, the impact of life disruptions on mental health disappeared at T2. Living standards decreased the proportions of mental health problems only at T1, which showed the negative association between socioeconomic status and mental health (41). Those with a low socioeconomic status might have experienced greater difficulties during the COVID-19 pandemic, such as a decreased income due to quarantine and a poor living environment. Our results might illustrate that sociodemographic factors mainly have short-term effects on the mental health outcomes of COVID-19 survivors, and mental health workers should pay more attention to those who suffer more from COVID-19 and have low socioeconomic status.

### Individual Factors Related to Mental Health Outcomes

Inconsistent with previous findings (2, 27), social support and BMI were not significant in the logistic regressions. Social support might mainly have an effect in the acute stage when comfort from friends and relatives could reduce the pandemic-related stress and loneliness of patients (42). An effect of BMI on mental health was not shown in the COVID-19 survivors. Further studies are needed to explore the effect of these factors in different samples and at different timepoints.

Higher resilience reduced the likelihood of depression only at T1, in line with previous reports (43, 44). However, the protective effect of resilience vanished at T2. Considering the drop in resilience between T1 and T2, a further study on the change in resilience and long-term resilience effects on mental health in COVID-19 survivors is needed.

Personality also affects mental health (20). Higher neuroticism was a risk factor for the three mental health outcomes at both T1 and T2, showing a stable long-term effect. Agreeableness had protective effects against the three mental health outcomes at T2; extraversion had protective effects against anxiety at T1 and T2; and openness had a protective effect against PTSD, depression, and anxiety at T1 and depression at T2. The results regarding the Big Five traits and mental health were in line with a previous study (20). Neuroticism was a key predictor for mental health problems, as individuals who are high in neuroticism experience a more negative affect and higher affective variability in their daily lives (45). Thus, psychological workers should pay special attention to those with high neuroticism. Nikčević et al. reported that agreeableness and extraversion played key protective roles in mental health (20), as these two traits might increase social activities, and our findings supported this idea. Furthermore, we discovered that extraversion mainly had effects on anxiety. The possible reason might have been that those who are high in extraversion increase their support seeking and decrease their support provisions when facing threats (46) and are more likely to perceive received social support (47). Further research can be done on the association of extraversion and anxiety. While agreeableness mainly had effects at 12 months postdiagnosis, this might be because those who are high in agreeableness tend to both seek and provide support (46) and might not obtain much relief in the short term.

### Temporal Associations Related to Mental Health Outcomes

Consistent with previous studies (21, 23), we discovered that PTSD scores at T1 could predict PTSD symptoms at T2; depression scores at T1 could predict depression symptoms at T2, which was inconsistent with studies during the COVID-19 pandemic (20, 22); and there was no predictive effect of anxiety. COVID-19 survivors might develop PTSD symptoms after discharge, especially as the pandemic continues to persist, and they might suffer discrimination and social exclusion (48) or other negative impacts caused by the disease. Mental health workers could provide some interventions to prevent chronic PTSD symptoms and pay more attention to those individuals with PTSD symptoms. The inconsistent predictive effects of depression and anxiety may be related to several factors. First, as the pandemic was gradually controlled in China, the panic and anxiety of survivors gradually decreased, which could have reduced follow-up mental health problems. Second, anxiety might increase one’s information seeking (49), which reduces uncertainty about the pandemic and self-health. Third, depression might reduce one’s social and daily activities (50), causing depression symptoms to worsen. Further studies should explore the temporal associations among these mental health symptoms during the COVID-19 pandemic in larger and broader samples. Mental health workers could implement interventions for those with high levels of PTSD or depressive symptoms to prevent long-term mental health problems.

### Limitations and Implications

There are some limitations of this study. First, in this study, data were obtained from self-report questionnaires; clinical diagnoses could be used in the future. Second, the sample for the current study was only from China and was not large and representative; future studies should include a more diverse sample of participants, such as patients from different countries. Third, some important factors, such as fear of reinfection (51) and pandemic prevention burnout (52), were not assessed in the current study but might have had negative effects on the mental health of the survivors of COVID-19. These factors should be assessed in future studies.

There are several important implications for psychological interventions as a result of our findings. First, we revealed that the mental health of COVID-19 survivors improved between 6 and 12 months postdiagnosis. Second, due to the much longer duration of the COVID-19 pandemic compared to previous pandemics, survivors may exhibit mental health problems in the long term, and mental health workers should continue to follow the mental health status of discharged COVID-19 patients over the long term. Third, our results indicated that at different timepoints, the predictors of mental health may vary; risk factors included higher neuroticism, increased PTSD and depression symptoms at T1, greater life disruptions caused by COVID-19, and poorer living standards, while protective factors included higher openness, extraversion, agreeableness, and resilience. Mental health workers should pay more attention to those with more risk factors and help to promote protective factors.

## Data Availability Statement

The raw data supporting the conclusions of this article will be made available by the authors on reasonable request.

## Ethics Statement

The studies involving human participants were reviewed and approved by Ethics Review Committee of the Institute of Psychology, Chinese Academy of Sciences. The patients/participants provided their written informed consent to participate in this study.

## Author Contributions

All authors listed have made a substantial, direct, and intellectual contribution to the work, and approved it for publication.

## Conflict of Interest

The authors declare that the research was conducted in the absence of any commercial or financial relationships that could be construed as a potential conflict of interest.

## Publisher’s Note

All claims expressed in this article are solely those of the authors and do not necessarily represent those of their affiliated organizations, or those of the publisher, the editors and the reviewers. Any product that may be evaluated in this article, or claim that may be made by its manufacturer, is not guaranteed or endorsed by the publisher.
